# CoCryoViS: Collaborative online cryo‐electron tomography visualization system

**DOI:** 10.1002/pro.70679

**Published:** 2026-07-10

**Authors:** Omar Mena, Uroš Šmajdek, Weiping Zhang, Piao Yu, Tobias Klein, Stefan T. Arold, Sai Li, Ivan Viola, Ciril Bohak

**Affiliations:** ^1^ King Abdullah University of Science and Technology Thuwal Kingdom of Saudi Arabia; ^2^ Faculty of Computer and Information Science University of Ljubljana Ljubljana Slovenia; ^3^ Tsinghua University School of Life Sciences Bejing China; ^4^ Nanographics Gmbh Wien Austria

**Keywords:** collaborative visualization, CryoET, segmentation, tomographic reconstruction, volume visualization, WASM, WebGPU

## Abstract

We present a collaborative, interactive, web‐based visualization and processing system for Cryo‐Electron Tomography (cryo‐ET) data that supports the complete workflow, from raw tilt‐series projections to final 3D segmentations. The system integrates tilt‐series alignment, motion correction, tomographic reconstruction, manual and pseudo‐annotation, deep model training, inference, and visualization, all accessible through a unified web‐based interface. The backend manages computationally intensive tasks, including advanced tomographic reconstruction, pseudo‐annotation generation, deep learning model training, and large‐scale segmentation of new datasets. It features separate queues for CPU and GPU tasks, allowing users to prepare and manage large workloads in advance. The frontend supports lightweight tomographic reconstruction, manual data annotation, segmentation with pretrained models, interactive exploration of pipeline stages, and real‐time monitoring of queued tasks. Key processing steps are available on both the client and server sides, providing flexibility for diverse computational environments, enabling visualization of externally hosted datasets, and supporting data privacy when required. The system is designed for collaborative workflows, enabling multiple users to share input data, annotations, and trained models within joint projects. Its modular architecture allows easy extension with additional stages on either the client or server side, making it adaptable to specialized pipelines within individual institutions. We demonstrate the system's capabilities on representative cryo‐ET datasets, highlighting its utility for both individual researchers and distributed teams working on structural analysis of complex cellular environments. The initial release of the system is available at https://cocryovis.lgm.fri.uni-lj.si/ (Project repository: https://github.com/nanovis/cocryovis, mirror deploy: https://tomography.kaust.edu.sa/), and the entire system will become open source so that any visualization or domain researcher can further enhance its functionality.

## 1 INTRODUCTION

Cryo‐electron tomography (cryo‐ET) has emerged as a go‐to imaging method for studying biological structures in their native environments. It enables the visualization of complex cellular architectures, large viral assemblies, bacteria, and subcellular components at near‐native conditions, providing unique insights that bridge the gap between structural and cell biology (Zheng & Cai, 2024). Prominent use cases include imaging of SARS‐CoV‐2 viral particles, bacterial ultrastructures, and cellular environments for understanding macromolecular interactions in situ. While not as widely utilized as the standard cryogenic electron microscopy (cryo‐EM) imaging, this modality is being used more frequently as a uniquely bridging modality between structural biology, concerned with the nanoscale detail, and cell biology, studying structures roughly at the microscale level.

The standard cryo‐ET workflow begins with the acquisition of Tilted Projection Images (Tilt‐Series), followed by motion correction, contrast transfer function (CTF) estimation, and Tilt‐Series alignment. These 2D projections are then combined into a 3D tomogram through tomographic reconstruction. Downstream analysis involves particle picking or segmentation, followed by subtomogram averaging to reach high resolution. However, a primary challenge in cryo‐ET is the extremely low signal‐to‐noise ratio (SNR) inherent in these reconstructions. This primarily arises due to the limited electron dose required to prevent radiation damage, detective quantum efficiency of the camera, and signal modulation by the CTF. Additionally, the missing wedge artifact caused by limited tilt range during acquisition further degrades volume quality.

Current software solutions address various stages of this pipeline. For preprocessing and reconstruction, IMOD (Mastronarde & Held, 2017) remains a foundational player, offering automated tilt‐series alignment and reconstruction. EMAN2 (Chen et al., 2017; Tang et al., 2007) provides a full pipeline including automatic alignment, while AreTomo (Zheng et al., 2022) offers an integrated package for marker‐free, motion‐corrected alignment. More recently, Warp and WarpTools (Tegunov et al., 2026) provide integrated tomographic reconstruction workflows from raw frames. For high‐throughput processing, TomoNet (Wang et al., 2024) and ScipionTomo (Jiménez de la Morena et al., 2022) focus on integration, reproducibility, and validation. RELION (Burt et al., 2024; Scheres, 2012) has also evolved into an integrated cryo‐ET framework supporting workflows from raw data to subtomogram averaging, while Dynamo (Castaño‐Díez et al., 2012) remains widely adopted for flexible subtomogram analysis and scripting in high‐performance computing (HPC) environments.

Particle picking and segmentation have seen rapid advancement through deep learning. While early tools like TomoSegMemTV (Martinez‐Sanchez et al., 2014) focused on robust membrane detection, newer deep learning pickers such as crYOLO (Wagner et al., 2019), TomoTwin (Rice et al., 2023), DeepFinder (Moebel et al., 2021) and PickYOLO (Genthe et al., 2023) have emerged. Complementary template‐matching approaches include template matching in PyTom (Hrabe et al., 2012), PyTME (Maurer et al., 2024), and the STOPGAP framework (Wan et al., 2020), including its GPU‐accelerated Python implementation GAPSTOP™ (Cruz‐León et al., 2024). TomoNet specifically addresses the challenge of picking on flexible lattices and surfaces (Wagner & Raunser, 2025). For structural heterogeneity, tools like cryoDRGN‐ET (Rangan et al., 2024) and the recently introduced OPUS‐TOMO (Luo et al., 2024) allow for the reconstruction of dynamic biomolecules. Despite these advances, visualization is often delayed until the final stages of the pipeline. Standard 3D rendering fails to differentiate biological signals from noise, although our prior work (Nguyen et al., 2022) showed that segmentation‐guided visualization can make early exploration feasible.

Web‐based tools are also gaining traction; for instance, nextPYP provides a comprehensive and scalable platform for the full pipeline. In the domain of visualization and interactive analysis, ArtiaX (Ermel et al., 2022) and UCSF ChimeraX (Pettersen et al., 2021) have introduced sophisticated handling of subtomograms and structures. However, many existing tools are desktop‐based or lack seamless collaboration features.

For the first time, we aim to integrate visualization and collaboration together with artificial intelligence methods into the cryo‐ET discovery process through **C**ollaborative **O**nline **Cryo**‐ET **Vi**sualization **S**ystem (CoCryoViS). Our system allows users not only to visualize volumes but also to perform reconstruction, sparse annotations, and trigger model training. The presented web‐based client–server system democratizes 3D visualization and AI‐assisted segmentation. Its modular architecture allows researchers to adapt the system to institutional pipelines. In this paper, we highlight the following contributions:

1. A modular and extensible platform that integrates interactive visualization across all stages of the cryo‐ET processing pipeline, from tomogram preprocessing to deep learning‐based segmentation inference.
2. A collaborative pipeline architecture that enables multiple users to contribute simultaneously to shared projects, either by working in parallel or sequentially across different stages of the workflow.
3. Dual‐mode execution for selected processing steps, supporting both client‐side and server‐side computation depending on available hardware resources and data privacy requirements.
4. Built‐in project sharing capabilities, including link‐based access with configurable permissions, to facilitate collaboration, reproducibility, dissemination, and public engagement.

The rest of the paper is structured as follows: In the following section (Section 2), we discuss related work and highlight the gaps our research addresses. We demonstrate and discuss our system through three use cases in Sections 3 and 4. We summarize our work and outline possible future work in Section 5. In Section 6, we describe the entire pipeline along with its individual stages, and present the implementation details.

## 2 RELATED WORK

The development of CoCryoViS is situated within a landscape of specialized tools for cryo‐ET data processing and visualization. While many individual stages of the pipeline are well supported by standalone software, the integration of these stages into a collaborative, web‐based environment remains a significant gap.

### 2.1 End‐to‐end processing frameworks

A major milestone in the field was the introduction of IMOD (Mastronarde et al., 1996; Mastronarde & Held, 2017), which through its *eTomo* graphical user interface was the first to offer a comprehensive, step‐by‐step workflow for tomographic processing from alignment to reconstruction. Similarly, EMAN2 (Chen et al., 2017; Tang et al., 2007) provides a full‐stack environment for cellular cryo‐ET, incorporating neural networks for automated annotation. While these desktop‐based tools are foundational, they are primarily designed for single‐user local execution. More recently, frameworks like ScipionTomo (Jiménez de la Morena et al., 2022), RELION (Burt et al., 2024; Scheres, 2012), Warp/WarpTools (Tegunov et al., 2026) and nextPYP (Liu et al., 2023) have addressed the need for integration and scalability. WarpTools extends processing from raw frame alignment through tomographic reconstruction, while RELION supports integrated refinement and subtomogram averaging workflows. nextPYP, in particular, shares our goal of providing a web‐based, scalable platform for characterizing protein variability in situ, though CoCryoViS places a heavier emphasis on interactive, segmentation‐guided 3D visualization during the early processing stages.

### 2.2 Advanced visualization and interaction

Visualization in cryo‐ET is dominated by high‐performance desktop applications. UCSF ChimeraX (Pettersen et al., 2021) and its toolbox extension ArtiaX (Ermel et al., 2022) provide sophisticated environments for the interactive handling and visualization of sub‐tomograms and molecular structures. In the domain of volumetric exploration and annotation, *napari* (Chiu & Clack, 2022) has emerged as a versatile ecosystem, with plugins like *blik* (Gaifas et al., 2024) extending its capabilities to cryo‐ET data visualization and analysis. Other specialized tools include Tomviz (Schwartz et al., 2022), which enables real‐time 3D analysis during data acquisition. While these tools offer high visual fidelity and precise interaction, they generally lack the collaborative project‐sharing mechanisms and the unified client–server task management provided by CoCryoViS.

### 2.3 AI‐driven segmentation and heterogeneity

Automated segmentation is essential for interpreting noisy tomograms. Beyond early solutions like TomoSegMemTV (Martinez‐Sanchez et al., 2014) for membrane detection, contemporary pipelines such as MemBrain (Lamm et al., 2022) and SynapseNet (Muth et al., 2025) offer specialized deep learning models for cellular components. General‐purpose tools like Ilastik (Berg et al., 2019) have also become standard for interactive machine learning in bioimage analysis. Complementary to learning‐based approaches, template‐matching frameworks including PyTME (Maurer et al., 2024), template matching in PyTom (Hrabe et al., 2012), and GAPSTOP™ (Cruz‐León et al., 2024) provide efficient strategies for detecting macromolecular complexes in tomographic volumes. In parallel, generative modeling tools such as cryoDRGN‐ET (Rangan et al., 2024) and OPUS‐TOMO (Luo et al., 2024) have advanced the field by resolving structural and compositional heterogeneities. CoCryoViS complements these efforts by integrating Ilastik‐based pseudo‐annotation and neural segmentation directly into the visualization workflow, allowing users to iteratively refine models and share them within a joint project.

### 2.4 Collaborative infrastructure

The transition to facility‐scale processing has prompted the development of collaborative pipelines. Systems implemented at facilities like eBIC (Horstmann et al., 2024) manage large‐scale data acquisition, while frameworks described by Baldwin et al. (2018) and Mendez et al. (2023) focus on automated evaluation. However, as noted by Isenberg et al. (2011), effective collaborative visualization requires addressing shared state and distributed interaction. Existing solutions are often limited to institutional clusters; CoCryoViS addresses this by utilizing a modern web stack (WebGPU/WASM) to deliver high‐performance, collaborative 3D visualization directly in the browser, regardless of the user's location.

Existing pipelines and tools offer robust capabilities across preprocessing, reconstruction, segmentation, and visualization. However, they are often constrained by several factors. There is a lack of unified, web‐based systems that seamlessly integrate all stages of the cryo‐ET processing workflow. Collaborative workflows and shared project management features remain limited. No single platform currently offers both lightweight client‐side processing and high‐performance server‐side computation. Moreover, visualization solutions are often fragmented and lack integration with annotation, segmentation, and model training functionalities.

Our proposed system addresses these limitations by providing a collaborative, modular, and extensible web‐based environment. It integrates tomographic reconstruction, manual and pseudo‐annotation, model training, segmentation, and interactive visualization, with support for both client‐ and server‐side execution. Furthermore, it enables collaborative workflows with shared datasets, annotations, and trained models, making it adaptable for both individual researchers and distributed teams.

## 3 RESULTS

In order to evaluate and illustrate the capabilities of CoCryoViS, we applied the system to three distinct cryo‐ET datasets spanning different biological targets and scales. Each use case (detailed in Sections 3.1 to 3.3) demonstrates the integrated workflow and highlights qualitative outcomes such as segmentation clarity, system performance, and collaborative user experience.

### 3.1 SARS‐CoV‐2: Full workflow demonstration

To showcase the complete downstream workflow assisted by CoCryoViS, we processed an in‐house cryo‐ET dataset containing SARS‐CoV‐2 virions, originally acquired for the study by Yao et al. (2020) and is also available at EMDB.^1^ The dataset was collected on a Titan Krios microscope operated at 300 kV, equipped with a Gatan K3 detector and a GIF Quantum energy filter (20 eV slit). Tilt‐series were recorded in super‐resolution mode at a pixel size of 0.68 Å using a dose‐symmetric scheme from −60° to +60° at 3° steps, with a total dose of 131.2 e^−^/Å^2^. The tomogram used in CoCryoViS has a grid size of 1024 × 1440 × 360 voxels with a voxel size of 5.44 Å (binned from the original acquisition). Initially, we loaded the tomogram capturing several virus particles into the system (Figure 3, top left). We manually annotated representative regions using CoCryoViS tools to isolate virus particles from the background, generating sparse segmentation masks (Figure 3, top right). Next, using these manual annotations, we employed the integrated Ilastik‐based (Berg et al., 2019) pseudo‐labeling module (see also Section 6.1.3). This step expanded our sparse annotations across the entire volume, producing a dense probabilistic mask that effectively captured additional viral structures not explicitly labeled in the previous step (Figure 3, middle left). After manually labeling a set of tomograms and generating the corresponding pseudo‐labels, we trained a 3D U‐Net + ResNet deep learning model directly on these pseudo‐labeled volumes. After training, we applied the model to the existing tomogram, yielding a refined segmentation mask (Figure 3, middle right). Such a trained model can be used for direct segmentation of new tomograms without the need for additional manual and/or pseudo‐annotation. Finally, we performed volumetric rendering of the pseudo‐annotations, and the inference‐based segmentation masks highlighted individual virions and structural features, improving interpretability compared to the tomogram (Figure 3, bottom left and bottom right). This streamlined workflow demonstrates CoCryoViS's capability to seamlessly transition between stages—from tomogram annotation through automatic segmentation and interactive visualization.

### 3.2 Vimentin filaments: Pseudo‐labeling and structure clarity

The second use case highlights the precision of our system on a medium‐sized in‐house generated tomogram containing elongated, thin vimentin intermediate fibroblast cells treated with detergent. The tomogram has a grid size of 4096 × 4096 × 61 voxels with a voxel size of 1.19 Å. The identity of the filaments was confirmed through subtomogram averaging analysis, which revealed a diameter consistent with vimentin (~10 nm) rather than F‐actin (~7 nm). To initiate the segmentation process, the user placed 15 annotation strokes along representative filaments across four slices, supplemented by three background annotations on separate slices, as shown in Figure 4. These sparse annotations were then propagated throughout the volume using the pseudo‐labeling module integrated with Ilastik. The result is a comprehensive segmentation mask that successfully captures the majority of visible actin filaments without requiring exhaustive manual labeling. This strategy reduces the annotation workload while providing a reliable approximation of filamentous structures.

A qualitative evaluation of the resulting segmentation revealed accurate identification of individual vimentin strands, preserving their continuity in 3D space.

### 3.3 Parainfluenza virus: Scalability and collaboration

The third use case demonstrates CoCryoViS's capability to process large tomograms, using a publicly available dataset of Human Parainfluenza Virus Fusion Complex Glycoproteins (EMPIAR‐10476)^2^ shown in Figure S1, Supporting Information. The dataset comprised two tomograms in MRC format, each with a grid size of 1919 × 1855 × 436 voxels and a pixel size of 3.68 Å. The total upload size was approximately 5.8 GB, selected specifically to evaluate the system's scalability and performance when handling high‐resolution data.

Only minimal manual annotation was required: the user marked the outline of a representative viral particle and selected a corresponding background region as can be seen in Figures S2, S3. We then initiated pseudo‐label propagation using integrated adaptation of the Ilastik software (Berg et al., 2019) shown in Figures S4, S5. Although this step took several hours due to the dataset's size, it ran asynchronously via the server's queuing system. This allowed users to continue interacting with the dataset by exploring different regions, adjusting visualization settings, refining annotations, or switching to another volume entirely, all without interrupting the ongoing processing task.

This use case also demonstrates the collaborative strengths of CoCryoViS. Once the project was shared, multiple users were able to instantly access the dataset, annotations, and intermediate results. The processed volume was concurrently visualized from three separate client machines on the same network, demonstrating the server's ability to efficiently handle multiple visualization requests. This enabled team members to provide immediate feedback without the need to transfer large files between local devices, streamlining collaborative analysis and communication.

### 3.4 Processing times

Across our experiments, we manually annotated approximately 50 SARS‐CoV‐2, 5 parainfluenza, and 5 vimentin tomograms. Manual annotation of a single tomogram in CoCryoViS typically required 5–15 min depending on volume size and structural complexity. Pseudo‐label generation via Ilastik scaled roughly linearly with voxel count: for SARS‐CoV‐2 volumes at 448 × 512 × 512 voxels, the random forest classifier trained in under 1 min and inference completed in approximately 4 min; at 448 × 1024 × 1024, these times increased to approximately 14 min and 2.2 h, respectively. The vimentin dataset (4096 × 4096 × 61, approximately 1 billion voxels) required approximately 2.7 h for classifier training. The parainfluenza dataset (1919 × 1855 × 436, approximately 1.5 billion voxels) required approximately 4.9 h for classifier training and approximately 2.5 h for inference. All pseudo‐labeling tasks ran asynchronously via the server queue, allowing users to continue working during processing. Neural network inference with the trained 3D Residual U‐Net was substantially faster across all datasets: under 3 s for small SARS‐CoV‐2 volumes (448 × 256 × 256), approximately 1 min for larger SARS‐CoV‐2 volumes (448 × 1024 × 1024), and approximately 3–4 min for the vimentin and parainfluenza datasets. Once trained, the model can segment new tomograms in seconds to minutes, eliminating the need to repeat the computationally expensive pseudo‐labeling pipeline for each new volume.

## 4 DISCUSSION

### 4.1 System limitations

While the presented system incorporates a wide range of functionalities, several limitations remain that we aim to address in future iterations. Domain scientists have highlighted the value of integrating multiple state‐of‐the‐art methods into each pipeline stage, enabling direct comparisons within a unified environment. This could be supported by extending the visualization module to allow simultaneous rendering of multiple datasets for qualitative assessment.

Although the current rendering system performs efficiently and supports real‐time interaction, there is potential to further enhance visual fidelity. Incorporating advanced techniques such as volumetric path tracing could allow for the generation of high‐quality, production‐grade stills suitable for publication and dissemination.

One of the limitations of our system is also the fiducial‐based alignment of the tilt‐series. This would require an extension of the user interface with several additional functionalities which we plan as part of the future work.

At the infrastructure level, the system is designed to execute on a single compute node (i.e., a single physical or virtual machine) equipped with multiple GPUs. However, the current implementation does not provide native support for distributed multi‐node execution or cluster‐level scheduling, although it can be deployed on a cluster node assuming appropriate container runtime and resource configuration. Extending it with distributed execution mechanisms (e.g., MPI, job schedulers such as Slurm, or container orchestration frameworks like Kubernetes) would enable horizontal scalability, reduce execution time, and improve throughput under concurrent workloads.

Finally, the system currently supports direct data fetching exclusively from the Cryo‐ET Data Portal. It does not support other commonly used repositories with less standardized formats, such as EMPIAR,^3^ limiting data source coverage. Addressing this limitation would broaden the applicability and streamline the data processing pipelines.

### 4.2 Expert evaluation and future directions

To gather qualitative insights into the system's utility and usability, CoCryoViS was presented and demonstrated during three venues: the 2025 *Tsinghua Spring School on Cryo‐ET with Dynamo*, a research group in the *Biological and Environmental Sciences program* at KAUST, and the Chan Zuckerberg Imaging Institute (CZII) journal club. Across all settings, feedback was consistently positive, with participants highlighting the value of rapid visualization, intuitive collaborative workflows, and the significant reduction in annotation time compared to conventional tools such as Amira‐Avizo, where slice‐by‐slice manual annotation of large structures can require up to 3 days. To the best of our knowledge Amira‐Aviso does not support sparse labeling and generation of pseudo labels for segmentation model training. The web‐based nature of CoCryoViS was particularly well received, as it eliminates the need for SSH connections or remote desktop configurations, lowering the barrier to entry for teams operating under strict institutional network policies. Unfortunately, we were not able to compare the proposed pipeline with the DragonFly (Heebner et al., 2022) system which is well accepted in the community.

Beyond general reception, domain users identified several concrete directions for future development. Recurring requests included the integration of additional reconstruction and preprocessing tools such as AreTomo (Zheng et al., 2022) and DeepDeWedge (Wiedemann et al., 2024), support for volume cropping to focus on specific regions of interest, and batch processing capabilities for reproducible multi‐volume pipelines. Users also expressed interest in the ability to simultaneously display multiple datasets for direct qualitative comparison—a feature that would be particularly valuable for rapid assessment of reconstruction or segmentation quality across conditions. Additionally, the ability to load data directly from repositories such as EMPIAR was highlighted as a valuable extension, broadening the range of publicly available datasets that can be explored within the system. These suggestions reflect the broader need for flexible, modular systems that can adapt to diverse institutional pipelines, and they directly inform our planned extensions to CoCryoViS.

## 5 CONCLUSION

We have introduced CoCryoViS, a modular, web‐based system that bridges the cryo‐ET pipeline from raw tilt‐series to interactive segmentation and 3D real‐time visualization. Compared with conventional workflows that split the preprocessing, annotation, segmentation, and visualization into separate tools, CoCryoViS provides a unified and collaborative environment that suits both individual researchers and distributed teams.

CoCryoViS couples advanced visualization techniques with annotation‐aware segmentation that allows users to interactively explore noisy cryo‐ET datasets and derive biological insights far earlier in the discovery process by including pseudo‐labeling and semi‐supervised neural training, which significantly reduces the load of manual annotation while enabling robust model generalization across a variety of volumes.

Its dual‐mode client–server design allows for flexible computation while remaining accessible. Besides, its modular architecture allows for the smooth integration of emerging tools and current institutional pipelines.

In conclusion, CoCryoViS represents a significant initial step in democratizing and facilitating the cryo‐ET analysis workflow under one system by combining high‐performance backend computation along with an intuitive web‐based interface allowing researchers to start with raw Tilt‐Series data until biological insights. CoCryoViS' collaborative and extensible design makes it a practical solution for today's challenges in cryo‐ET and a strong foundation for future developments in visualization, annotation, and AI‐assisted analysis. With its future open‐source release, we aim to inspire a community of users and contributors to continue refining and expanding the system, driving innovation in structural biology at the cellular scale.

## 6 METHODS

### 6.1 System design

CoCryoViS is a client–server web application designed to streamline cryo‐ET data processing, covering the entire workflow from processing raw Tilt‐Series to final 3D volume segmentation and visualization, as illustrated in Figure 2. The system is designed to provide a powerful yet accessible browser‐based client interface for tasks such as data loading, manual annotation, inference, and visualization while offloading computationally intensive operations, including alignment, tomographic reconstruction, and neural model training, to a high‐performance remote server.

The system is structured around five distinct processing pipeline stages:

1. Preprocessing and tomographic reconstruction—Reconstructs the tomogram from raw Tilt‐Series data.
2. Manual annotation—Provides interactive tools for user‐guided labeling of structures.
3. Pseudo annotation—Generates dense probabilistic labels based on sparse manual annotations.
4. Neural segmentation—Consists of segmentation model training, which trains deep learning models using semi‐supervised neural learning approaches, and segmentation model inference, which applies trained models to predict segmentation on new data.
5. Visualization—Renders and enhances volumetric data for interactive analysis.

The following subsections describe each pipeline stage in detail. Users can use the pipeline stages sequentially to follow the entire processing workflow or individually by incorporating data preprocessed with external software.

To foster collaborative research, CoCryoViS is built with shared access mechanisms, allowing multiple users to collaborate on any pipeline stage. Datasets, trained models, and intermediate results are organized into projects, which can be shared with different permission levels, either read‐only or full‐write access, enabling efficient teamwork across research groups.

#### 6.1.1 Preprocessing and tomographic reconstruction

Cryo‐ET captures a series of 2D projection images from a sample at varying tilt angles along a defined axis, forming what is known as a Tilt‐Series. Due to the inherent limitations of individual projections, the images are seldom interpreted individually and are instead combined into a 3D volume (tomogram) through tomographic reconstruction as shown in Figure 5. This tomogram serves as a foundation for downstream processing, including segmentation, feature extraction, and visualization.

Before proceeding with tomographic reconstruction, raw Tilt‐Series images must be carefully aligned and preprocessed to correct various artifacts introduced during data collection. CoCryoViS implements three possible preprocessing steps using common preprocessing tools to streamline this process before the actual tomographic reconstruction:

1. Motion correction—Tilt‐Series images may exhibit frame misalignments, both globally and locally, caused by factors such as beam‐induced movement or stage drift. CoCryoViS, by default, integrates MotionCor3,^4^ an improved version of MotionCor2 by Zheng et al. (2017), which corrects motion artifacts by aligning image frames, thereby improving overall image sharpness and preserving high‐resolution information.
2. CTF estimation—After motion correction, the next step is typically to estimate the CTF. Accurate CTF estimation is critical for high resolution structural determination, particularly in subtomogram averaging workflows where preservation of the high resolution signal is essential (Turoňová et al., 2017). In CoCryoViS, GCtfFind^5^ is provided as a default option for this step, automatically detecting CTF parameters and providing feedback on data quality. While CTF correction may be less critical for tasks focused on contrast‐based visualization or segmentation, its inclusion ensures compatibility with diverse downstream workflows.
3. Tilt‐Series alignment—CoCryoViS integrates exclusively fiducial‐free, patch‐based IMOD (Mastronarde et al., 1996). The pipeline executes five binaries in sequence: ccderaser removes pixel‐level anomalies from the raw tilt series—specifically hot pixels (defective detector pixels reporting abnormally high values) and X‐ray events (bright spots caused by stray X‐rays striking the detector)—by identifying pixels whose intensity deviates sharply from their local neighborhood and replacing them with interpolated values (configurable peak and difference thresholds, defaults 10/10); note that the reprojection‐based gold bead erasing step of the standard IMOD pipeline is not applied, as CoCryoViS uses a fiducial‐free workflow; extracttilts reads per‐tilt angles from the MRC header; tiltxcorr performs patch‐based cross‐correlation tracking with configurable patch grid size (default 4), patch dimensions (default 680 px), and search radius (default 0.125 in relative units); tiltalign fits a single rigid transform per tilt image—no local deformation field is estimated—using the tracked correspondences; and newstack applies the computed transforms to produce the aligned stack. All IMOD output is captured in the task log accessible from the UI.

Given the critical role of tomographic reconstruction, CoCryoViS integrates two distinct approaches, both selected for their compatibility with GPU acceleration, aligning with the system's inherent GPU‐centric architecture. The first is Simultaneous Algebraic Reconstruction Technique (SART), an inexpensive approach that we have leveraged to allow the quick reconstruction on the user's client.

The second approach, a proximal jointly‐optimized method proposed by Ramirez et al. (2024), is deployed on the server, leveraging more computationally intensive optimization techniques (such as Total Variation) to achieve higher fidelity reconstructions. This dual‐strategy design allows users to decide between the efficiency of the basic reconstruction approach implemented directly in the browser and the accuracy of a more computationally expensive, but more time‐consuming approach available on the server, ensuring both on‐demand local processing and high‐quality results regardless of the client's computation capabilities. Finally, acknowledging that iterative and TV‐based methods are not suitable for high‐resolution subtomogram averaging workflows, CoCryoViS additionally provides a WBP (Weighted Back‐Projection) reconstruction method via IMOD's tilt, ensuring compatibility with downstream subtomogram averaging pipelines.

#### 6.1.2 Manual annotation

Once a user has either created or uploaded a 3D tomogram, CoCryoViS provides a robust manual annotation framework, enabling precise labeling of structures within the volume as depicted in Figure 6. Manual annotation is a crucial step in many workflows, serving purposes of training segmentation models, validating automated results, or highlighting key regions of interest for further analysis.

To make this process intuitive, we allow users to annotate from a familiar 2D perspective, using cutting planes. These include a view‐aligned plane and three orthogonal planes defined by the principal axes. By navigating slice‐by‐slice, users can accurately place annotations while maintaining a clear spatial understanding of the tomogram.

Annotations are represented as a kernel defined with weighted inverse distance and a configurable radius, forming a three‐dimensional annotation volume. Users retain complete control over positioning, rotation, and cutting plane to ensure an interactive and flexible annotation process. They can also toggle the visibility of other labels within the same tomogram as needed.

In CoCryoViS, annotations are stored on the server as single‐channel 3D volumes, enabling persistent, on‐demand editing throughout the analysis process. This design allows users to iteratively modify, refine, or extend existing annotations without disrupting the workflow, while also supporting straightforward export of annotation volumes as volume files for integration with external software tools or downstream pipelines. Through the system's sharing functionality, multiple users can collaboratively annotate the same project in parallel, facilitating coordinated analysis across distributed teams.

The amount of manual annotation required depends on the specific use case and desired segmentation accuracy. In practice, it is beneficial to distribute annotations across all three orthogonal planes, as this provides more robust spatial coverage and supports effective training of the used segmentation models.

#### 6.1.3 Pseudo annotation

Manual annotations are typically sparse, covering only a tiny fraction of the tomogram slices or volume. As noted by Nguyen et al. (2022), the crisp segmentation masks produced by manual annotations also do not accurately reflect the uncertainty resulting from the imaging modality. Their findings also indicate that even a weak segmentation algorithm, such as a random forest classifier, can effectively propagate user‐provided labels to other voxels within the volume, significantly extending the annotation coverage, as depicted in Figure 7.

Leveraging such an approach, we generate pseudo‐labels, which provide a dense probabilistic segmentation of the tomogram. These pseudo‐labels not only help experts infer additional structural details from the input data but can also be efficiently employed as part of a downstream semi‐supervised neural segmentation pipeline.

We employ the Pixel Classification workflow from the open‐source Ilastik software (Berg et al., 2019) in a headless mode, using a parallel Vigra Random Forest classifier with default tree count. Feature extraction uses Gaussian Smoothing (*σ* = 0.3, 1.0) and Gaussian Gradient Magnitude (*σ* = 1.0). Ilastik outputs per‐class probability maps as uint8 values (0–255, mapped from [0, 1]). These probabilities are not thresholded and are passed directly to the neural segmentation training stage. The input sparse manual annotations and the resulting pesudo‐labels are compared in Figure 4.

#### 6.1.4 Neural segmentation

While the pseudo‐annotation process effectively segments structures within a given tomogram based on sparse manual annotations, it does not generalize across multiple volumes. To address this limitation, CoCryoViS integrates the semi‐supervised neural segmentation pipeline proposed by Nguyen et al. (2022). as depicted in Figure 8. This pipeline is based on a modified 3D U‐Net + ResNet architecture (Lee et al., 2017), optimized for learning from probabilistic pseudo‐labeled volumes. The authors have shown that the approach is effective at performing two key segmentation tasks:

- Foreground‐background segmentation—the model learns to distinguish between the object of interest and the surrounding background,
- Class‐based segmentation—different biological structures within the tomogram are assigned distinct labels based on learned features.

Training a deep neural model for 3D segmentation is both computationally intensive and time‐consuming, requiring substantial system resources. To efficiently manage these demands, CoCryoViS restricts model training to the server‐side implementation, which is remotely accessible from user clients. This approach ensures that resource‐heavy operations are performed on dedicated hardware, allowing users to continue working on other tasks without maintaining an active client session. The resulting models can be stored either on the server directly or exported by the user to be employed in other software tools. On the other hand, inference, applying the trained model to new data, is computationally much less expensive than training. To maximize accessibility and efficiency, we have implemented dual inference support, enabling segmentation to be performed on both the client and the server:

- Client‐side inference—Users with capable GPUs can perform segmentation locally and on‐demand, ensuring fast results with minimal latency.
- Server‐side inference—Users without dedicated graphical hardware can still leverage the trained model via remote processing, ensuring broader accessibility.

Similar to annotations, the users can store the segmentation results on the server or save them directly on their hard drive to avoid sharing sensitive datasets. Alongside segmentation masks, CoCryoViS also generates an inverted, low‐pass filtered version of the reconstructed volume that can be used as part of the visualization pipeline. This preprocessing step emphasizes structural details within the volume, leveraging the fact that lower intensity values typically represent biological structures in cryo‐ET. The relation between the filtered reconstructed volume and visualization is further described in the following section.

#### 6.1.5 Visualization

**FIGURE 1:**
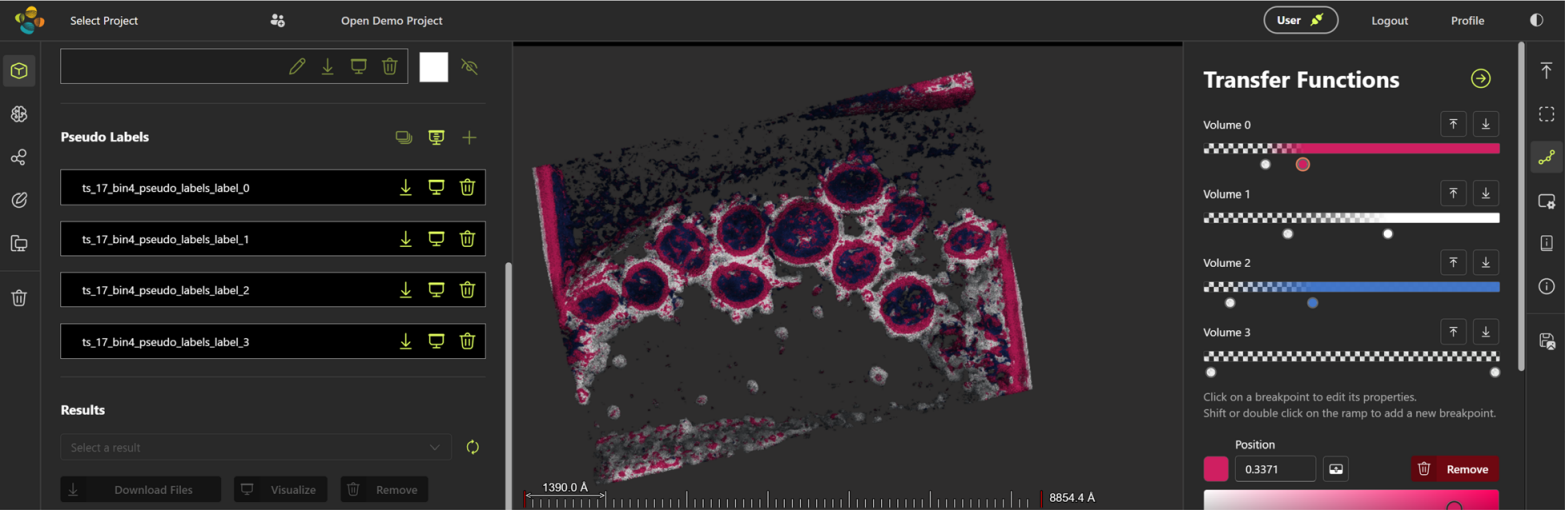
Our system visualizing generated pseudo labels for a selected tomogram (SARS‐CoV‐2 D614 strain; Yao et al., 2020) where users can visualize labels for individual or multiple classes (displayed in the left pane) simultaneously and define the visualization parameters (displayed in the right pane).

The visualization system employs volumetric ray casting implemented entirely in WebGPU compute shaders, combining front‐to‐back compositing with early ray termination, jittered sampling to suppress aliasing, local ambient occlusion (Hernell et al., 2010) for depth perception, and soft shadows (Veach, 1998) for light attenuation. These techniques are essential for cryo‐ET data, where the low signal‐to‐noise ratio and probabilistic segmentation outputs make subtle structural features difficult to distinguish without perceptual depth cues. Users can enable or disable individual class volumes, apply clipping planes, and adjust per‐class ramp‐based transfer functions (Figure 1, right pane). A ramp‐based design was chosen over conventional widget‐based transfer functions because it maps naturally to the probabilistic output of the segmentation stages and reduces the cognitive load when exploring noisy data. Advanced rendering parameters—sample rate, occlusion radius and strength, shadow quality—are exposed through a dedicated settings panel (Figure 9).

**FIGURE 2:**
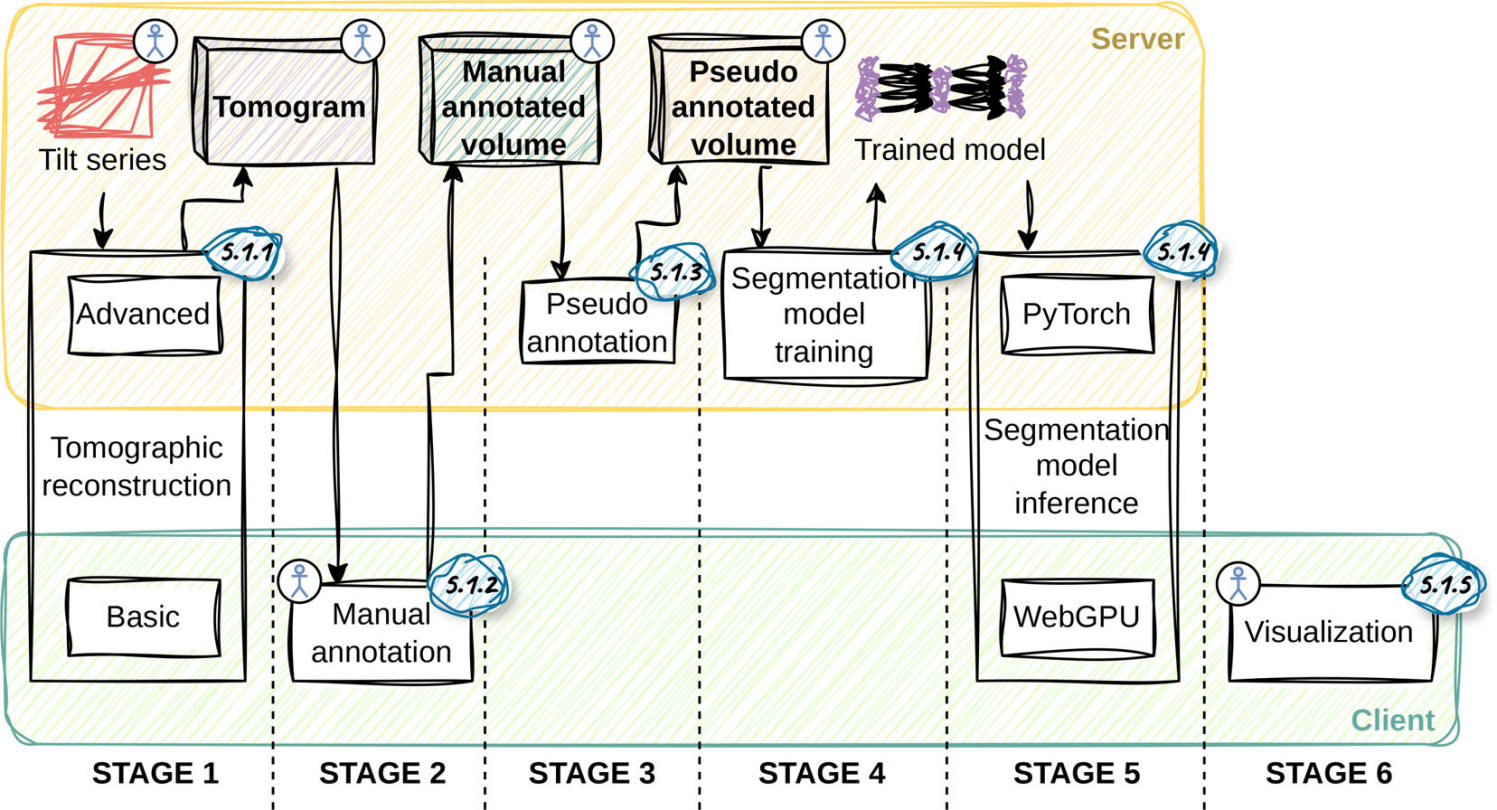
The overall system structure. User tags indicate the entry points where users can interact with the system or provide their own data.

**FIGURE 3:**
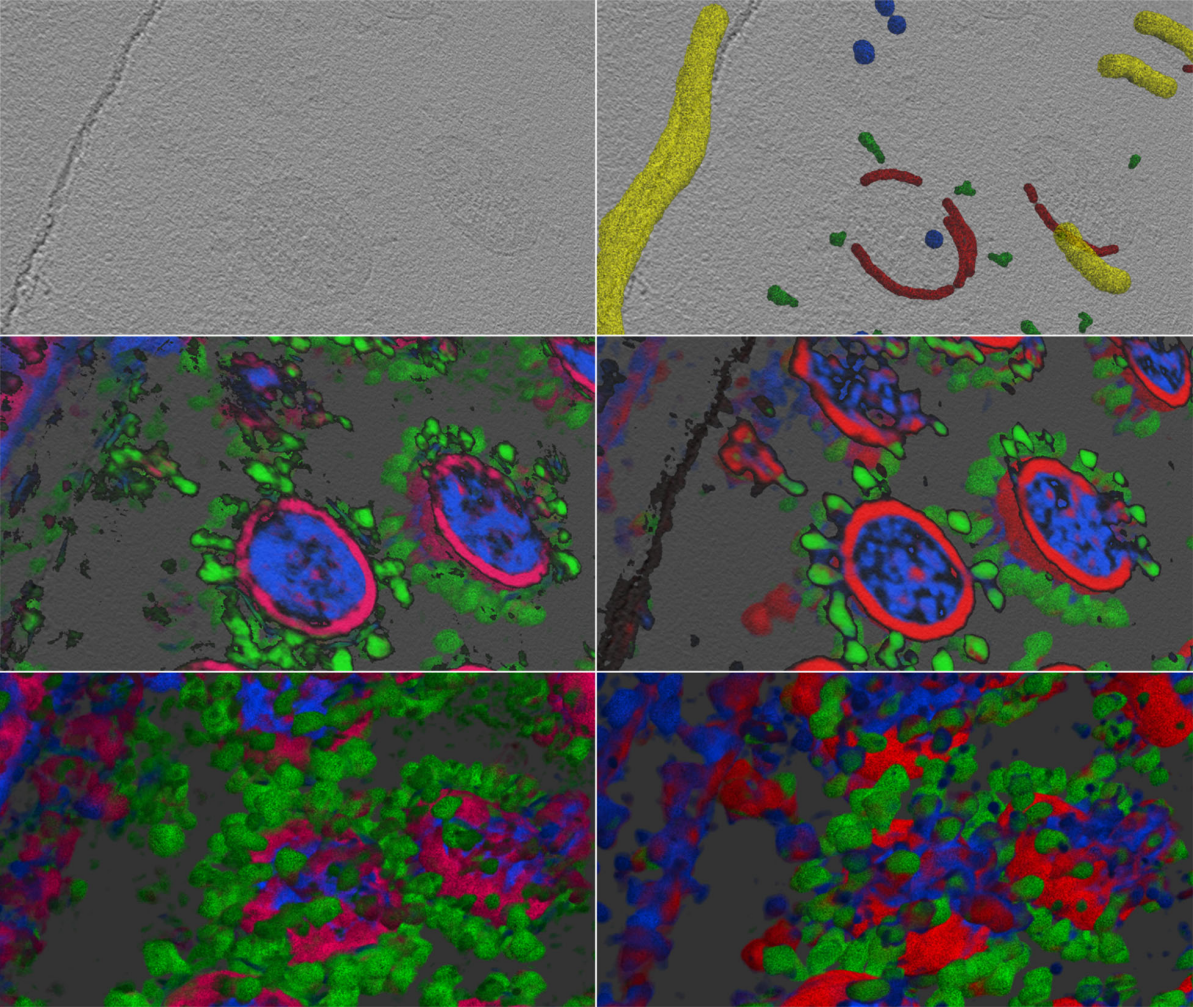
Top left: Cropped part of tomogram. Top right: Manual annotations. Middle left: Cropped view of pseudo‐annotations generated using Ilastik. Middle right: Cropped inference results using 3D U‐Net + ResNet model. Bottom left: Volume rendered pseudo‐annotations generated using Ilastik. Bottom right: Volume rendered inference results using 3D U‐Net + ResNet model. Individual classes are depicted using different colors: background (yellow) is only present in manual annotations, membrane (red), spike proteins (green), and lumen (blue).

**FIGURE 4:**
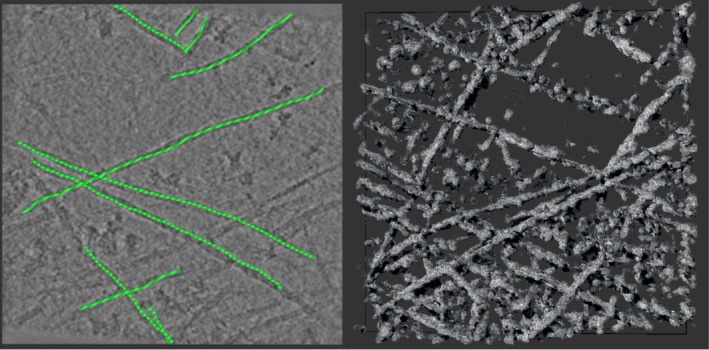
Left: Manual annotations placed by the user on representative vimentin filaments in a 2D slice from the 3D tomogram. Right: Resulting 3D pseudo‐label segmentation generated with Ilastik using annotations from the left image as an input. Both visualizations are visualized using CoCryoViS.

**FIGURE 5:**
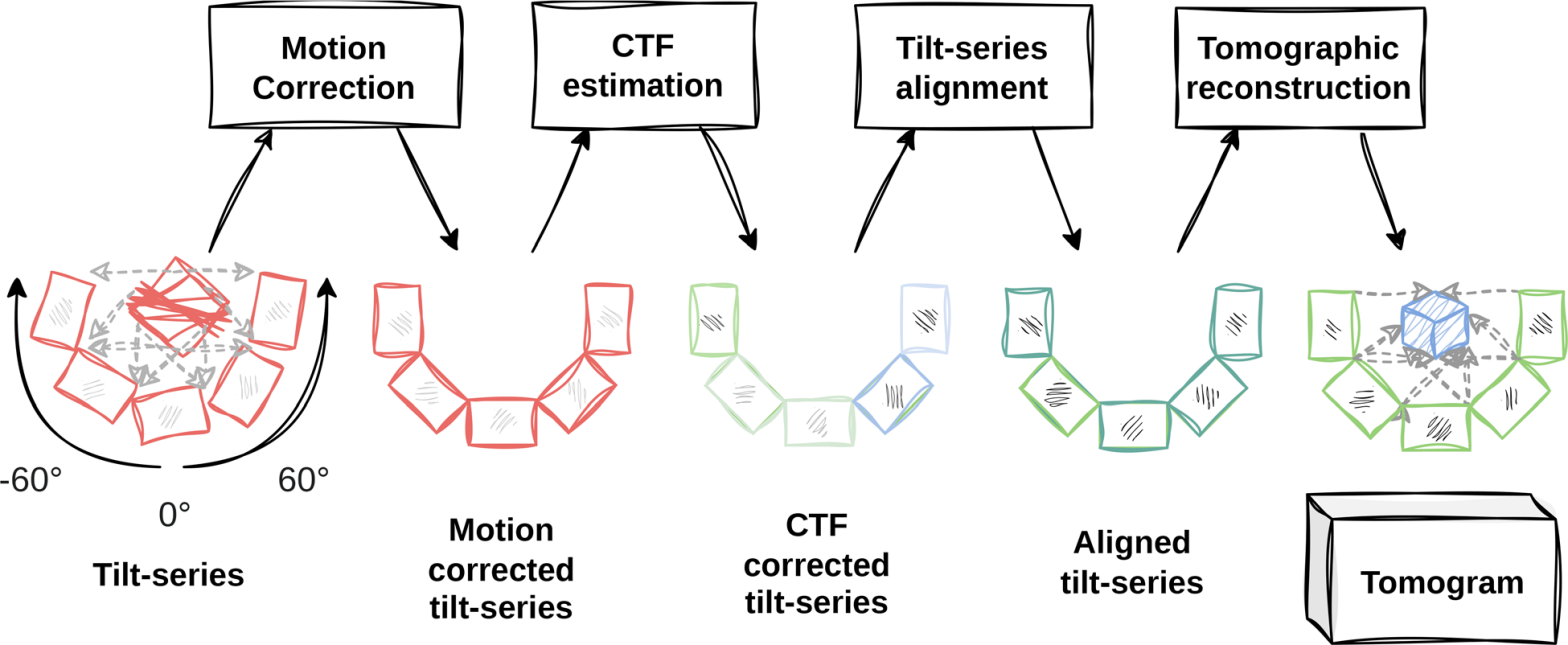
From tilt‐series alignment to tomographic reconstruction.

**FIGURE 6:**
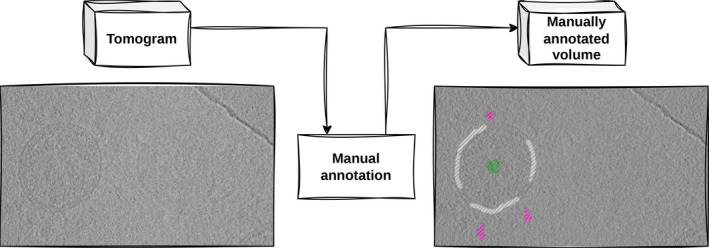
Visualization of manual annotation pipeline.

**FIGURE 7:**
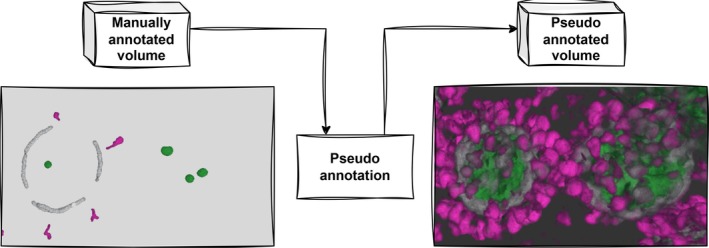
Visualization of pseudo annotation pipeline. For clarity, the annotation of the background label has been omitted.

**FIGURE 8:**
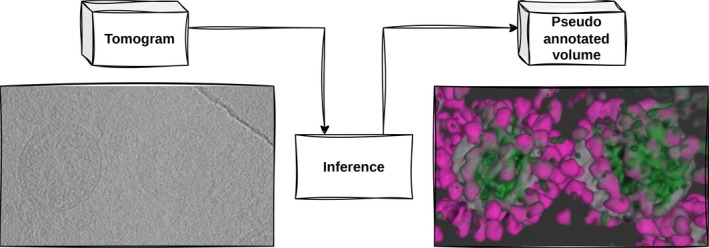
Visualization of neural training and segmentation results. For clarity, the annotation of the background label has been omitted. In the upper part of the left image, a volume rendering of pseudo labeled data is shown next to the original reconstructed volume in the bottom part of the left image. The right image shows the final segmented volume using a deep segmentation model.

**FIGURE 9:**
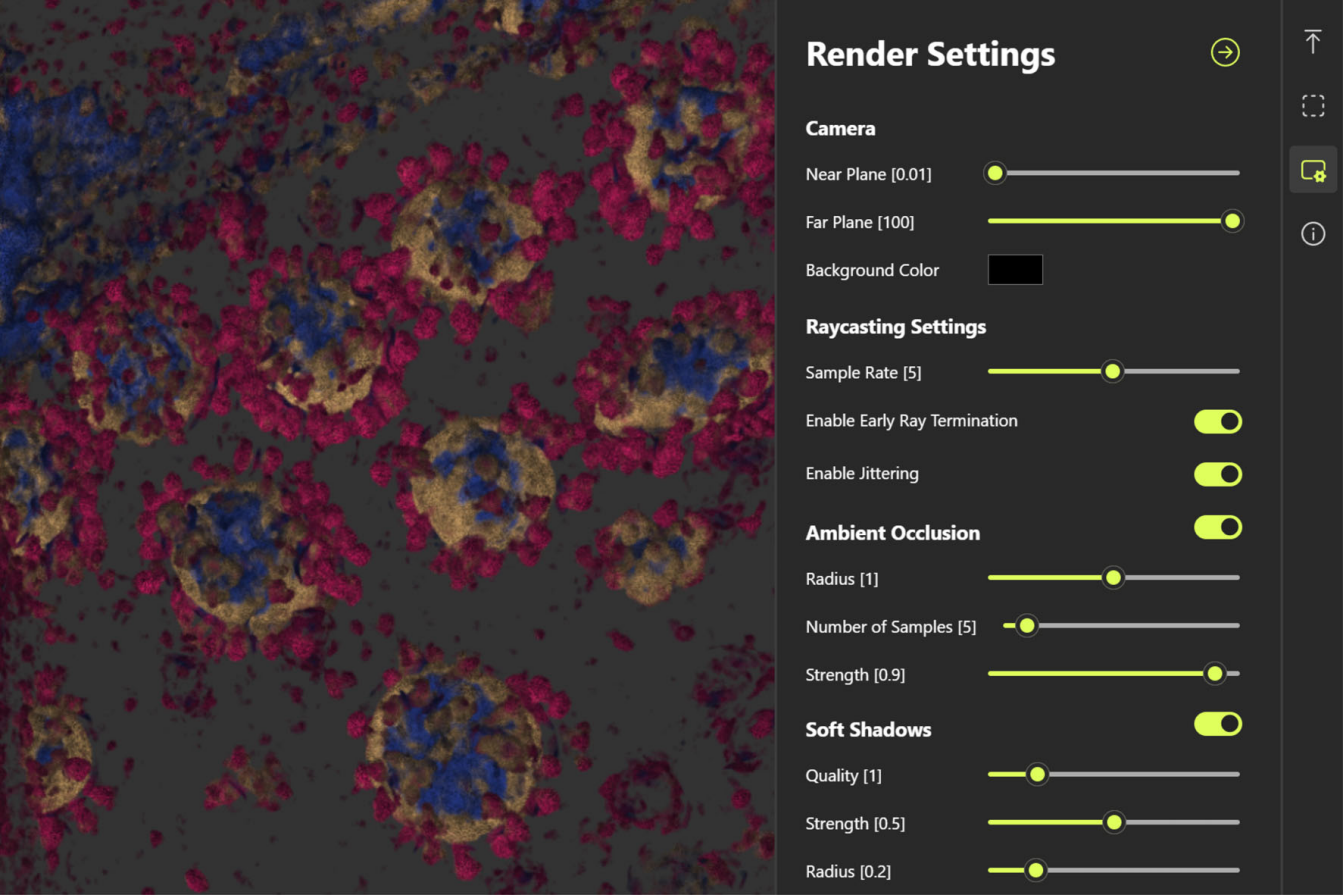
Rendering settings widget.

Volumetric visualization is available at every pipeline stage, adapted to the data produced so far, and is often coupled with slice visualization on a view‐ or axis‐aligned cutting plane. This joint rendering provides a sharp 2D cross‐section while preserving the lighting context of the surrounding volume.

### 6.2 Implementation

**FIGURE 10:**
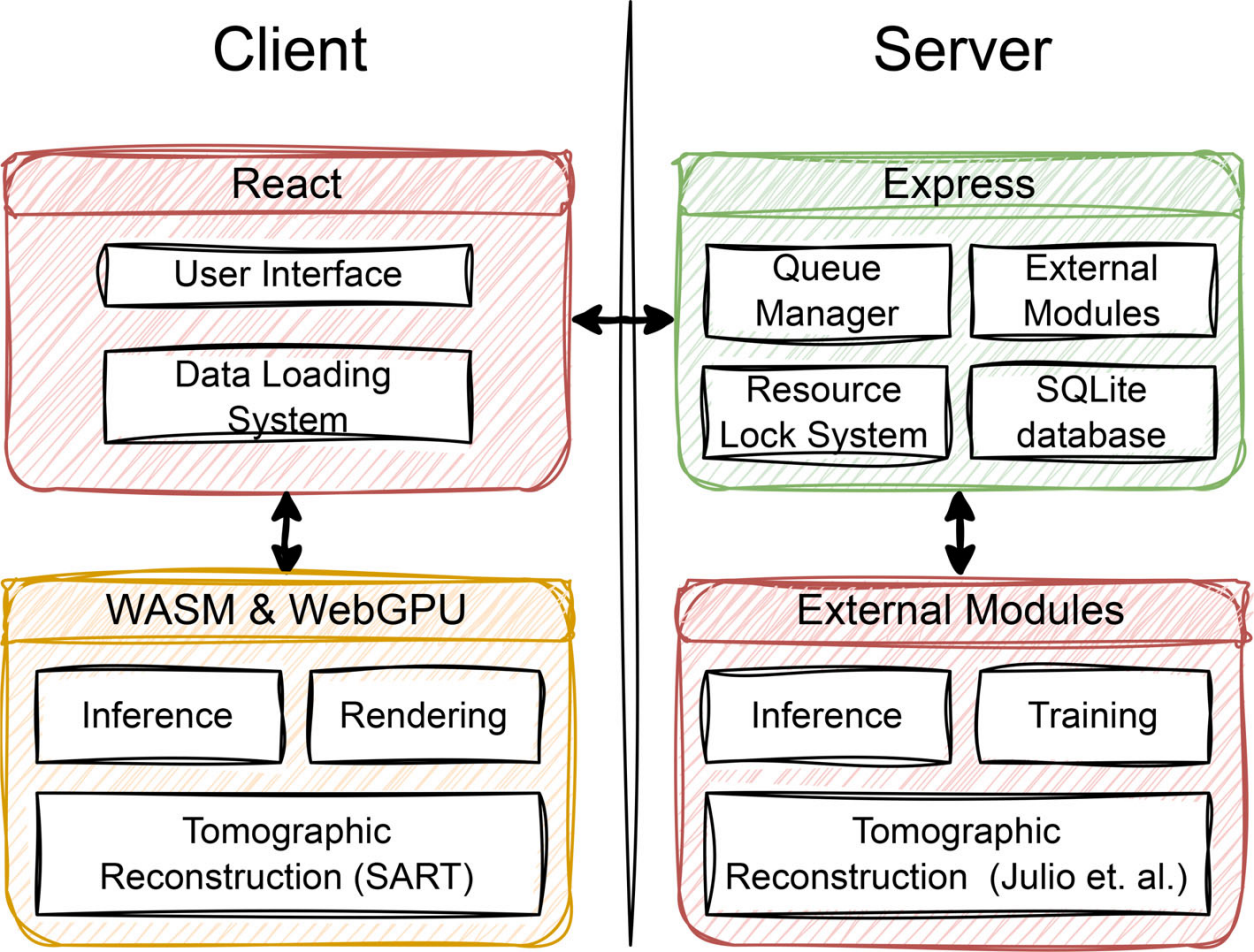
Schema of key components of the CoCryoViS implementation.

This section provides an overview of the technologies and architectural decisions behind the CoCryoViS. We will describe the key components of client and server implementations alongside the core technologies used for data processing, user interaction, and computational tasks, which are highlighted on Figure 10.

#### 6.2.1 Server

The CoCryoViS server manages several core functions, including handling API requests, interfacing with external modules, managing the queuing system, maintaining the internal database, and coordinating collaborative project access. To ensure long‐term maintainability and scalability, the server is built on the Node.js platform,^6^ utilizing the Express.js framework^7^ for API management. For ease of deployment, the system employs an SQLite database.^8^ However, it is designed with future adaptability in mind, using Prisma^9^ as an Object‐Relational Mapping (ORM) layer, allowing seamless migration to other database systems if needed.

##### Project storage and portability

Projects in CoCryoViS are stored directly on the server file system, with all associated data saved as raw files, while project metadata (e.g., structure definitions, processing states, and configuration parameters) are maintained in the database. The server is self‐contained and organized using relative file paths, the root of which can be configured via the system configuration. This design ensures that projects remain portable across different deployments, including containerized environments (e.g., Docker volumes), without requiring path reconfiguration. Although explicit versioning is not implemented, all processing steps are logged, providing traceability and supporting reproducibility.

##### Project sharing and synchronization

Collaborative access to projects is supported through a centralized server model. Multiple users can access and interact with the same project concurrently. Changes to project state are propagated in real time using WebSocket‐based communication, allowing connected clients to remain synchronized without requiring manual refresh.

##### Concurrent access and consistency

To ensure data consistency during concurrent usage, the system implements a locking mechanism at the structure level. Data entities involved in active processing tasks are temporarily locked to prevent modification, avoiding race conditions and ensuring reproducibility of results. Read access remains available, allowing users to inspect intermediate states while computations are in progress.

##### Task management

**FIGURE 11:**
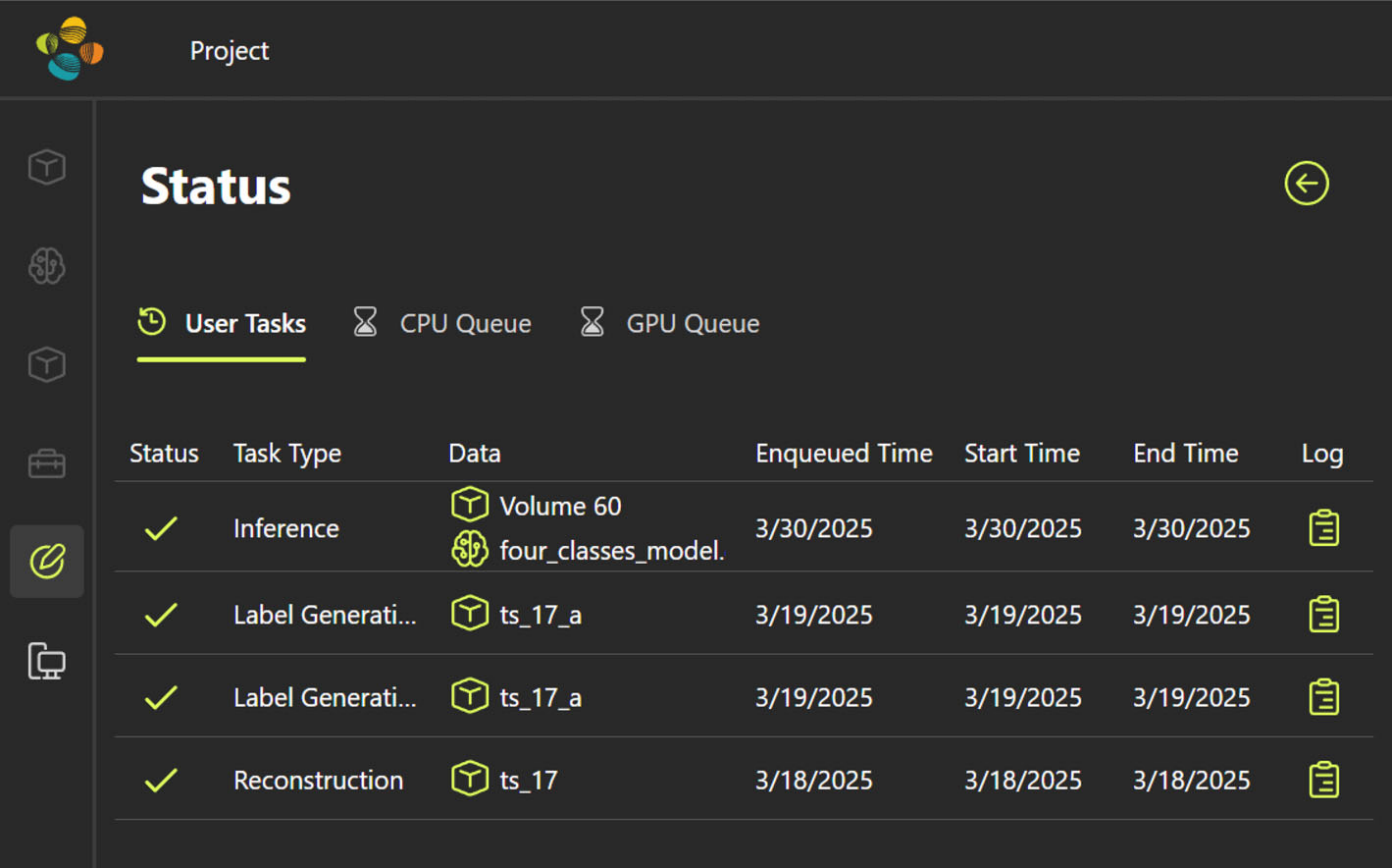
The Queuing System showing completed and failed tasks. Users can inspect the status of the individual task by clicking on the corresponding log icon on the right.

The queuing system ensures that computationally intensive tasks can be performed efficiently with respect to available server resources. When a server receives a complex task, such as neural model training or tomographic reconstruction, the task is placed into a designated queue. Currently, the system includes two separate queues: the CPU queue and the GPU queue. CPU queue processes a single active task at a time, while GPU queue can simultaneously utilize all available GPU's. Users can monitor the progress of queued and completed tasks in real time via the status widget, which also provides detailed logs for inspection and troubleshooting (Figure 11).

##### Extensibility

The system supports integration of external processing modules through a standardized interface, allowing users to define execution entry points and optional build procedures. This enables consistent incorporation of new modules into the queuing system and execution workflow. Integration into the broader application (e.g., API exposure and user interface) remains user‐defined, but can be achieved without substantial architectural changes by following existing implementation patterns. Currently supported modules include Ilastik (Berg et al., 2019), Nano‐Ötzi^10^ (Nguyen et al., 2022), Proximal CryoET^11^ (Ramirez et al., 2024), MotionCor3,^12^ GCtfFind^13^ and IMOD (Mastronarde et al., 1996). Additional details on the integration of external modules can be found in the project repository.^14^

##### Programmatic interface

All core system functionality is exposed through a RESTful API, enabling programmatic interaction with the server. This enables integration with external tools and scripting environments, supporting large‐scale and automated processing scenarios.

#### 6.2.2 Client

The CoCryoViS client serves two primary functions: it provides users with access to both client‐ and server‐side functionalities as described in Section 6 and interfaces with the WebGPU API^15^ for volumetric rendering, tomographic reconstruction, and neural segmentation. The client is built on the React framework,^16^ ensuring maintainability and a responsive user experience. The interface is constructed using Microsoft Fluent UI components,^17^ providing a consistent, accessible, and visually cohesive design.

The WebGPU API has been implemented within a 64‐bit WebAssembly framework using cross‐platform implementation Dawn,^18^ providing optimized memory management and near‐native performance for complex computations. Additionally, the client‐side tomographic reconstruction stage (using SART) and neural segmentation stage are implemented in WGSL^19^ compute shaders and are executed directly within the WebGPU compute pipeline, allowing for high‐performance execution without unnecessary data transfers between the CPU and GPU.

The client features an extensible data loading system, enabling users to upload data either to the client or server. Currently, the system supports three distinct data sources:

- Local upload—Users can select RAW and MRC files from their local filesystem. These files are preprocessed to extract metadata such as dimensions and data type. If the metadata is missing, users can manually input the required information.
- Remote URL—The client allows users to provide a URL linking to a remote repository where the dataset is stored. The system will then fetch and preprocess the data accordingly and allow the user to fill in the required metadata if it is missing in the source.
- Cryo‐ET data portal—In collaboration with the CZII,^20^ the client integrates with the Cryo‐ET data portal, allowing users to directly load tomograms using their unique dataset ID.

The client application includes support for executing selected pipeline stages locally, allowing users with high‐performance machines and dedicated GPUs to process data directly on their own systems. This not only bypasses the need to queue tasks on the server but also offers enhanced data privacy by keeping sensitive datasets on the user's machine.

### 6.3 Use of artificial intelligence generated content tools

The authors used Google Gemini and OpenAI ChatGPT to improve the grammar and language in the paper. The generated and corrected text was revised by the authors.

## AUTHOR CONTRIBUTIONS

**Tobias Klein:** Software; visualization. **Omar Mena:** Conceptualization; data curation; investigation; methodology; software; validation; writing – original draft; writing – review and editing. **Ivan Viola:** Conceptualization; funding acquisition; investigation; methodology; project administration; supervision; validation; writing – review and editing. **Stefan T. Arold:** Funding acquisition; investigation; resources; supervision; writing – review and editing. **Uroš Šmajdek:** Conceptualization; methodology; software; investigation; visualization; writing – original draft; writing – review and editing. **Ciril Bohak:** Conceptualization; methodology; software; data curation; investigation; validation; formal analysis; supervision; funding acquisition; visualization; project administration; writing – original draft; writing – review and editing. **Piao Yu:** Investigation; validation; writing – review and editing; data curation; resources. **Weiping Zhang:** Data curation; methodology; resources; validation; writing – review and editing. **Sai Li:** Formal analysis; funding acquisition; investigation; methodology; supervision; writing – review and editing.

## CONFLICT OF INTEREST STATEMENT

None of the authors have a conflict of interest to disclose.

## Supporting information


**Data S1.** Protein science 2025—Mena—Šmajdek—Supplementary material.pdf.

**Data S2.** Protein science 2025—Mena—Šmajdek—Supplemental Video.mp4. The video summarizes the system design, illustrates its functionalities and showcases its usage.

**Data S3.** Protein science 2025—Mena—Šmajdek—Supplemental Video.srt.

## Data Availability

Data sharing not applicable to this article as no datasets were generated or analysed during the current study.

## Footnotes

1
https://www.ebi.ac.uk/emdb/EMD-33297.

2
https://www.ebi.ac.uk/empiar/EMPIAR-10476/.

3
https://www.ebi.ac.uk/empiar/.

4
https://github.com/czimaginginstitute/MotionCor3.

5
https://github.com/czimaginginstitute/GCtfFind.

6
https://nodejs.org/.

7
https://expressjs.com/.

8
https://www.sqlite.org/.

9
https://www.prisma.io/.

10
https://github.com/nanovis/nano-oetzi.

11
https://github.com/juareyra/Proximal_CryoET/.

12
https://github.com/czimaginginstitute/MotionCor3.

13
https://github.com/czimaginginstitute/GCtfFind.

14
https://github.com/nanovis/cocryovis/blob/main/server/docs/modules-system.md.

15
https://www.w3.org/TR/webgpu/.

16
https://react.dev/.

17
https://github.com/microsoft/fluentui.

18
https://dawn.googlesource.com/dawn.

19
https://www.w3.org/TR/WGSL/.

20
https://www.czimaginginstitute.org/.

## References

[pro70679-bib-0001] Baldwin PR , Tan YZ , Eng ET , Rice WJ , Noble AJ , Negro CJ , et al. Big data in cryoEM: automated collection, processing and accessibility of EM data. Curr Opin Microbiol. 2018;43:1–8. 10.1016/j.mib.2017.10.005 29100109 PMC6067001

[pro70679-bib-0002] Berg S , Kutra D , Kroeger T , Straehle CN , Kausler BX , Haubold C , et al. Ilastik: interactive machine learning for (bio) image analysis. Nat Methods. 2019;16(12):1226–1232. 31570887 10.1038/s41592-019-0582-9

[pro70679-bib-0003] Burt A , Toader B , Warshamanage R , von Kügelgen A , Pyle E , Zivanov J , et al. An image processing pipeline for electron cryo‐tomography in RELION‐5. FEBS Open Bio. 2024;14(11):1788–1804. 10.1002/2211-5463.13873 PMC11532982 39147729

[pro70679-bib-0004] Castaño‐Díez D , Kudryashev M , Arheit M , Stahlberg H . Dynamo: a flexible, user‐friendly development tool for subtomogram averaging of cryo‐EM data in high‐performance computing environments. J Struct Biol. 2012;178(2):139–151. 10.1016/j.jsb.2011.12.017 22245546

[pro70679-bib-0005] Chen M , Dai W , Sun SY , Jonasch D , He CY , Schmid MF , et al. Convolutional neural networks for automated annotation of cellular cryo‐electron tomograms. Nat Methods. 2017;14(10):983–985. 10.1038/nmeth.4405 28846087 PMC5623144

[pro70679-bib-0006] Chiu C‐L , Clack N , the napari community . Napari: a python multi‐dimensional image viewer platform for the research community. Microsc Microanal. 2022;28(S1):1576–1577. 10.1017/S1431927622006328

[pro70679-bib-0007] Cruz‐León S , Majtner T , Hoffmann PC , Kreysing JP , Kehl S , Tuijtel MW , et al. High‐confidence 3D template matching for cryo‐electron tomography. Nat Commun. 2024;15(1):3992. 10.1038/s41467-024-47839-8 38734767 PMC11088655

[pro70679-bib-0008] Ermel UH , Arghittu SM , Frangakis AS . ArtiaX: an electron tomography toolbox for the interactive handling of sub‐tomograms in UCSF ChimeraX. Protein Sci. 2022;31(12):e4472. 10.1002/pro.4472 36251681 PMC9667824

[pro70679-bib-0009] Gaifas L , Kirchner MA, Timmins J , Gutsche I . Blik: an extensible 3D visualization tool for the annotation and analysis of cryo‐electron tomography data. PLOS Biology 22(4): e3002447. 2024. 10.1371/journal.pbio.3002447 38687779 PMC11268629

[pro70679-bib-0010] Genthe E , Miletic S , Tekkali I , James RH , Marlovits TC , Heuser P . PickYOLO: fast deep learning particle detector for annotation of cryo electron tomograms. J Struct Biol. 2023;215(3):107990. 10.1016/j.jsb.2023.107990 37364763

[pro70679-bib-0011] Heebner JE , Purnell C , Hylton RK , Marsh M , Grillo MA , Swulius MT . Deep learning‐based segmentation of Cryo‐electron tomograms. J Vis Exp. 2022;189:e64435. 10.3791/64435 36440884

[pro70679-bib-0012] Hernell F , Ljung P , Ynnerman A . Local ambient occlusion in direct volume rendering. IEEE Trans Vis Comput Graph. 2010;16(4):548–559. 10.1109/TVCG.2009.45 20467054

[pro70679-bib-0013] Horstmann A , Riggs S , Chaban Y , Clare DK , de Freitas G , Farmer D , et al. A service‐based approach to cryoEM facility processing pipelines at eBIC. Acta Crystallogr D Struct Biol. 2024;80(3):174–180. 10.1107/S2059798324000986 38376453 PMC10910546

[pro70679-bib-0014] Hrabe T , Chen Y , Pfeffer S , Cuellar LK , Mangold A‐V , Förster F . PyTom: a python‐based toolbox for localization of macromolecules in cryo‐electron tomograms and subtomogram analysis. J Struct Biol. 2012;178(2):177–188. 10.1016/j.jsb.2011.12.003 22193517

[pro70679-bib-0015] Isenberg P , Elmqvist N , Scholtz J , Cernea D , Ma K‐L , Hagen H . Collaborative visualization: definition, challenges, and research agenda. Inf Vis. 2011;10(4):310–326. 10.1177/1473871611412817

[pro70679-bib-0016] Jiménez de la Morena J , Conesa P , Fonseca YC , de Isidro‐Gómez FP , Herreros D , Fernández‐Giménez E , et al. ScipionTomo: towards cryo‐electron tomography software integration, reproducibility, and validation. J Struct Biol. 2022;214(3):107872. 10.1016/j.jsb.2022.107872 35660516 PMC7613607

[pro70679-bib-0017] Lamm L , Righetto RD , Wietrzynski W , Pöge M , Martinez‐Sanchez A , Peng T , et al. MemBrain: a deep learning‐aided pipeline for detection of membrane proteins in Cryo‐electron tomograms. Comput Methods Prog Biomed. 2022;224:106990. 10.1016/j.cmpb.2022.106990 35858496

[pro70679-bib-0018] Lee K , Zung J , Li P , Jain V , Seung HS . Superhuman accuracy on the SNEMI3D Connectomics challenge. arXiv Preprint. 2017:1–11.

[pro70679-bib-0019] Liu H , Zhou Y , Huang Q , Liu H‐F , Piland J , Jin W , et al. nextPYP: a comprehensive and scalable platform for characterizing protein variability in situ using single‐particle cryo‐electron tomography. Nat Methods. 2023;20:1909–1919. 10.1038/s41592-023-02045-0 37884796 PMC10703682

[pro70679-bib-0020] Luo Z , Chen X , Wang Q , Ma J . OPUS‐TOMO: towards resolving dynamics and compositional heterogeneities of biomolecules with cryo‐electron tomography. bioRxiv. 2024:1–81. 10.1101/2024.06.30.601442

[pro70679-bib-0021] Martinez‐Sanchez A , Garcia I , Asano S , Lucic V , Fernandez JJ . Robust membrane detection based on tensor voting for electron tomography. J Struct Biol. 2014;186(1):49–61. 10.1016/j.jsb.2014.02.015 24625523

[pro70679-bib-0022] Mastronarde DN , Held SR . Automated tilt series alignment and tomographic reconstruction in IMOD. J Struct Biol. 2017;197(2):102–113. 10.1016/j.jsb.2016.07.011 27444392 PMC5247408

[pro70679-bib-0023] Mastronarde DN , Kremer JR , McIntosh JR . Computer visualization of three‐dimensional image data using IMOD. J Struct Biol. 1996;116:71–76. 10.1006/jsbi.1996.0013 8742726

[pro70679-bib-0024] Maurer VJ , Siggel M , Kosinski J . PyTME (python template matching engine): a fast, flexible, and multi‐purpose template matching library for cryogenic electron microscopy data. SoftwareX. 2024;25:101636.

[pro70679-bib-0025] Mendez JH , Chua EYD , Paraan M , Potter CS , Carragher B . Automated pipelines for rapid evaluation during cryoEM data acquisition. Curr Opin Struct Biol. 2023;83:102729. 10.1016/j.sbi.2023.102729 37988815

[pro70679-bib-0026] Moebel E , Martinez‐Sanchez A , Lamm L , Righetto RD , Wietrzynski W , Albert S , et al. Deep learning improves macromolecule identification in 3D cellular cryo‐electron tomograms. Nat Methods. 2021;18(11):1386–1394. 10.1038/s41592-021-01275-4 34675434

[pro70679-bib-0027] Muth S , Moschref F , Freckmann L , Mutschall S , Hojas‐Garcia‐Plaza I , Bahr JN , et al. SynapseNet: deep learning for automatic synapse reconstruction. Molecular Biology of the Cell. 2025;36(10):1–19. 10.1091/mbc.E24-11-0591 PMC12483319 40875337

[pro70679-bib-0028] Nguyen N , Bohak C , Engel D , Mindek P , Strnad O , Wonka P , et al. Finding Nano‐Ötzi: Cryo‐electron tomography visualization guided by learned segmentation. IEEE Trans Vis Comput Graph. 2022;28:1–18. 10.1109/TVCG.2022.3186146 35749328

[pro70679-bib-0029] Pettersen EF , Goddard TD , Huang CC , Meng EC , Couch GS , Croll TI , et al. UCSF ChimeraX: structure visualization for researchers, educators, and developers. Protein Sci. 2021;30(1):70–82. 10.1002/pro.3943 32881101 PMC7737788

[pro70679-bib-0030] Ramirez JR , Rautek P , Bohak C , Strnad O , Zhang Z , Li S , et al. GPU accelerated 3D tomographic reconstruction and visualization from Noisy electron microscopy tilt‐series. IEEE Trans Vis Comput Graph. 2024;30(7):3331–3345. 10.1109/TVCG.2022.3230445 37015451

[pro70679-bib-0031] Rangan R , Feathers R , Khavnekar S , Lerer A , Johnston JD , Kelley R , et al. CryoDRGN‐ET: deep reconstructing generative networks for visualizing dynamic biomolecules inside cells. Nat Methods. 2024;21(8):1537–1545. 10.1038/s41592-024-02340-4 39025970

[pro70679-bib-0032] Rice G , Wagner T , Stabrin M , Sitsel O , Prumbaum D , Raunser S . TomoTwin: generalized 3D localization of macromolecules in cryo‐electron tomograms with structural data mining. Nat Methods. 2023;20(6):871–880. 10.1038/s41592-023-01878-z 37188953 PMC10250198

[pro70679-bib-0033] Scheres SHW . RELION: implementation of a Bayesian approach to cryo‐EM structure determination. J Struct Biol. 2012;180(3):519–530. 10.1016/j.jsb.2012.09.006 23000701 PMC3690530

[pro70679-bib-0034] Schwartz J , Harris C , Pietryga J , Zheng H , Kumar P , Visheratina A , et al. Real‐time 3D analysis during electron tomography using tomviz. Nat Commun. 2022;13(1):4458. 10.1038/s41467-022-32046-0 35915070 PMC9343612

[pro70679-bib-0035] Tang G , Peng L , Baldwin PR , Mann DS , Jiang W , Rees I , et al. EMAN2: an extensible image processing suite for electron microscopy. J Struct Biol. 2007;157(1):38–46. 10.1016/j.jsb.2006.05.009 16859925

[pro70679-bib-0036] Tegunov D , Burt A , Chaillet M , Shah PNM , Janse MHA , et al. warpem/warp: v2.0.0dev39. Zenodo. 2026 10.5281/zenodo.20065201

[pro70679-bib-0037] Turoňová B , Schur FKM , Wan W , Briggs JAG . Efficient 3D‐CTF correction for cryo‐electron tomography using NovaCTF improves subtomogram averaging resolution to 3.4Å. J Struct Biol. 2017;199(3):187–195. 10.1016/j.jsb.2017.07.007 28743638 PMC5614107

[pro70679-bib-0038] Veach E . Robust monte carlo methods for light transport simulation. PhD thesis. Stanford, CA, USA: Stanford University; 1998.

[pro70679-bib-0039] Wagner T , Merino F , Stabrin M , Moriya T , Antoni C , Apelbaum A , et al. SPHIRE‐crYOLO is a fast and accurate fully automated particle picker for cryo‐EM. Commun Biol. 2019;2(1):218. 10.1038/s42003-019-0437-z 31240256 PMC6584505

[pro70679-bib-0040] Wagner T , Raunser S . Cryo‐electron tomography: challenges and computational strategies for particle picking. Curr Opin Struct Biol. 2025;93:103113. 10.1016/j.sbi.2025.103113 40639056

[pro70679-bib-0041] Wan W , Khavnekar S , Wagner J , Erdmann P , Baumeister W . STOPGAP: a software package for subtomogram averaging and refinement. Microsc Microanal. 2020;26(S2):2516. 10.1017/S143192762002187X

[pro70679-bib-0042] Wang H , Liao S , Yu X , Zhang J , Zhou ZH . TomoNet: a streamlined cryogenic electron tomography software pipeline with automatic particle picking on flexible lattices. Biol Imaging. 2024;4:e7. 10.1017/S2633903X24000060 38828212 PMC11140495

[pro70679-bib-0043] Wiedemann S , Fabian Z , Soltanolkotabi M , Heckel R . A deep learning method for simultaneous denoising and missing wedge reconstruction in cryogenic electron tomography. Nat Commun. 2024;15(1):8255. 10.1038/s41467-024-51438-y 39313517 PMC11420219

[pro70679-bib-0044] Yao H , Song Y , Chen Y , Wu N , Xu J , Sun C , et al. Molecular architecture of the SARS‐CoV‐2 virus. Cell. 2020;183(3):730–738.e13. 10.1016/j.cell.2020.09.018 32979942 PMC7474903

[pro70679-bib-0045] Zheng S , Wolff G , Greenan G , Chen Z , Faas FGA , Bárcena M , et al. AreTomo: an integrated software package for automated marker‐free, motion‐corrected cryo‐electron tomographic alignment and reconstruction. J Struct Biol X. 2022;6:100068. 10.1016/j.yjsbx.2022.100068 35601683 PMC9117686

[pro70679-bib-0046] Zheng S , Palovcak E , Armache J‐P , Verba KA , Cheng Y , Agard DA . MotionCor2: anisotropic correction of beam‐induced motion for improved cryo‐electron microscopy. Nat Methods. 2017;14(4):331–332. 10.1038/nmeth.4193 28250466 PMC5494038

[pro70679-bib-0047] Zheng T , Cai S . Recent technical advances in cellular cryo‐electron tomography. Int J Biochem Cell Biol. 2024;175:106648. 10.1016/j.biocel.2024.106648 39181502

