# Supplementary material for: CoCryoViS: Collaborative online cryo‐electron tomography visualization system
Source: Protein Sci. 2026 Jul 10;35(8):e70679. doi: 10.1002/pro.70679 (PMC13354744; doi:10.1002/pro.70679)
Supplement: Supplementary file 1 — Data S1. Protein science 2025—Mena—Šmajdek—Supplementary material.pdf. [file PRO-35-e70679-s003.pdf]

CoCryoViS: Collaborative Online CryoET Visualization System

— Supplementary Material —

Omar Mena[1], Uroš Šmajdek[2], Weiping Zhang[3], Piao Yu[3], Tobias Klein[4], Stefan Arold[1], Sai Li[3], Ivan Viola[1], Ciril Bohak[1,2]

shared first authorship

corresponding author

[1] King Abdullah University of Science and Technology, Thuwal, Kingdom of Saudi Arabia

[2] Faculty of Computer and Information Science, University of Ljubljana, Ljubljana, Slovenia

[3] Tsinghua University School of Life Sciences, Bejing, China

[4] Nanographics Gmbh, Wien, Austria

1 User Manual

For detailed instructions on how to use the different tools available with this dataset, please check the Demo project[1], where you can also find the link to the user manual in the right-hand toolbar of the system.

2 Use Case: Parainfluenza Virus

This section provides supplementary figures illustrating the Human Parainfluenza Virus Fusion Complex Glycoproteins dataset to highlight its structural characteristics.

[1]accessible at:
`https://cocryovis.lgm.fri.uni-lj.si/demo`
`https://tomography.kaust.edu.sa/demo`

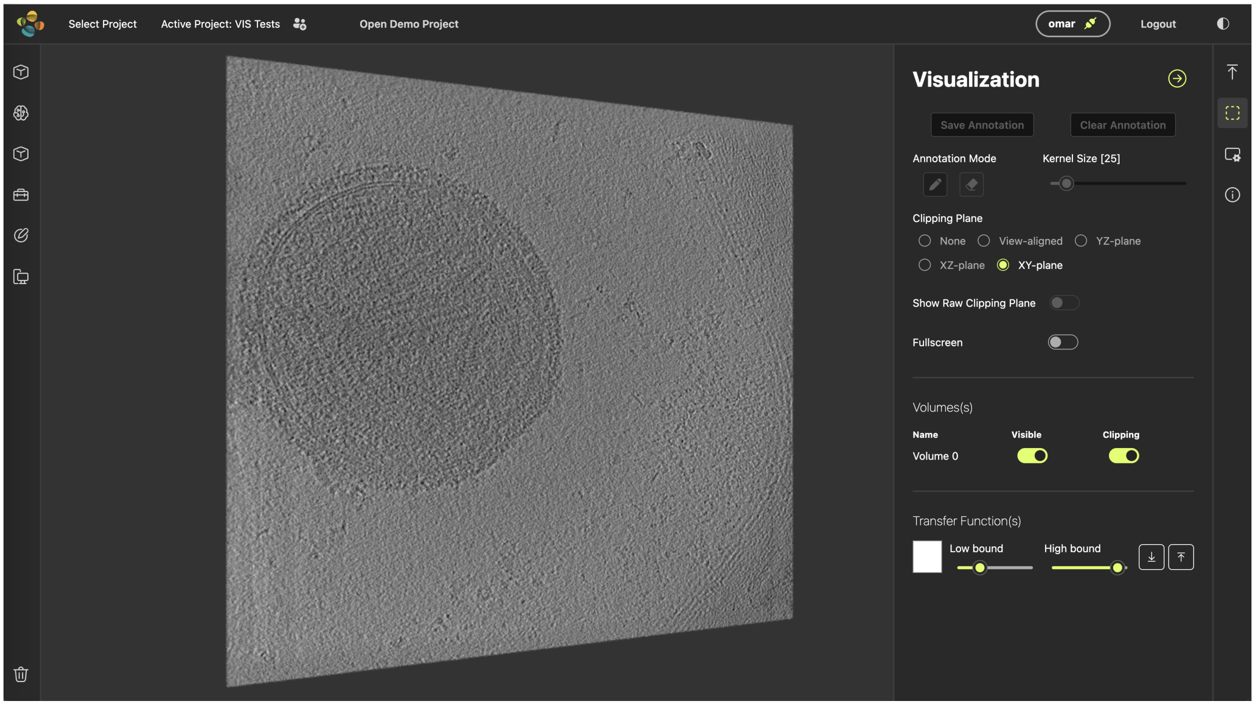

Figure 1: Parainfluenza Virus angled face

2.1 Large volume support

The dataset comprises a tomogram measuring 1919 1855 436 voxels in 32-bit float precision, resulting in a file size of approximately 5.8 GB. This large volume emphasizes the necessity for scalable visualization approaches and robust data-handling strategies. We conducted the visualization and rendering as shown in ?? using Firefox Nightly on a MacBook Pro M4 with 48 GB of RAM. This setup effectively handled the memory-intensive rendering demands, confirming CoCryoViS's capability to manage and interactively display large tomograms at responsive frame rates.

2.2 Collaboration

A small group of three people from the Biological and Environmental Science program at KAUST performed a minimal manual annotation to differentiate between the virus as shown

in ?? and the background regions as shown in ??.

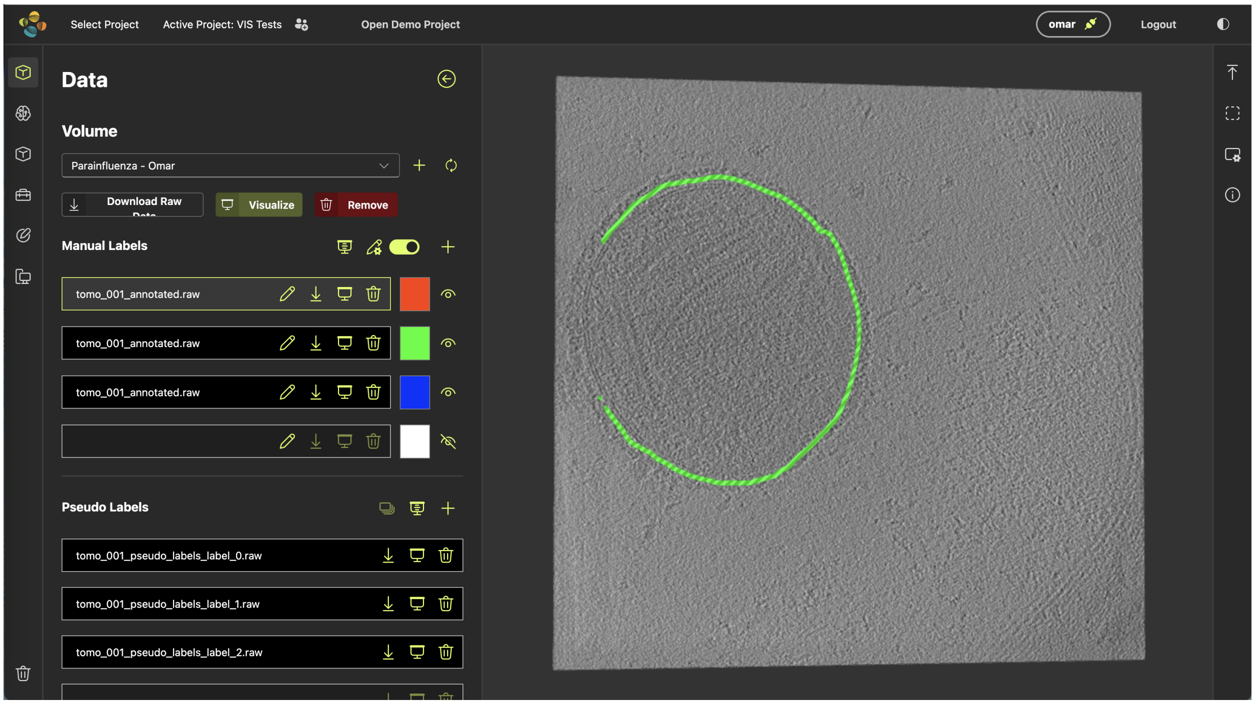

Figure 2: Manual annotation of Virus's outline

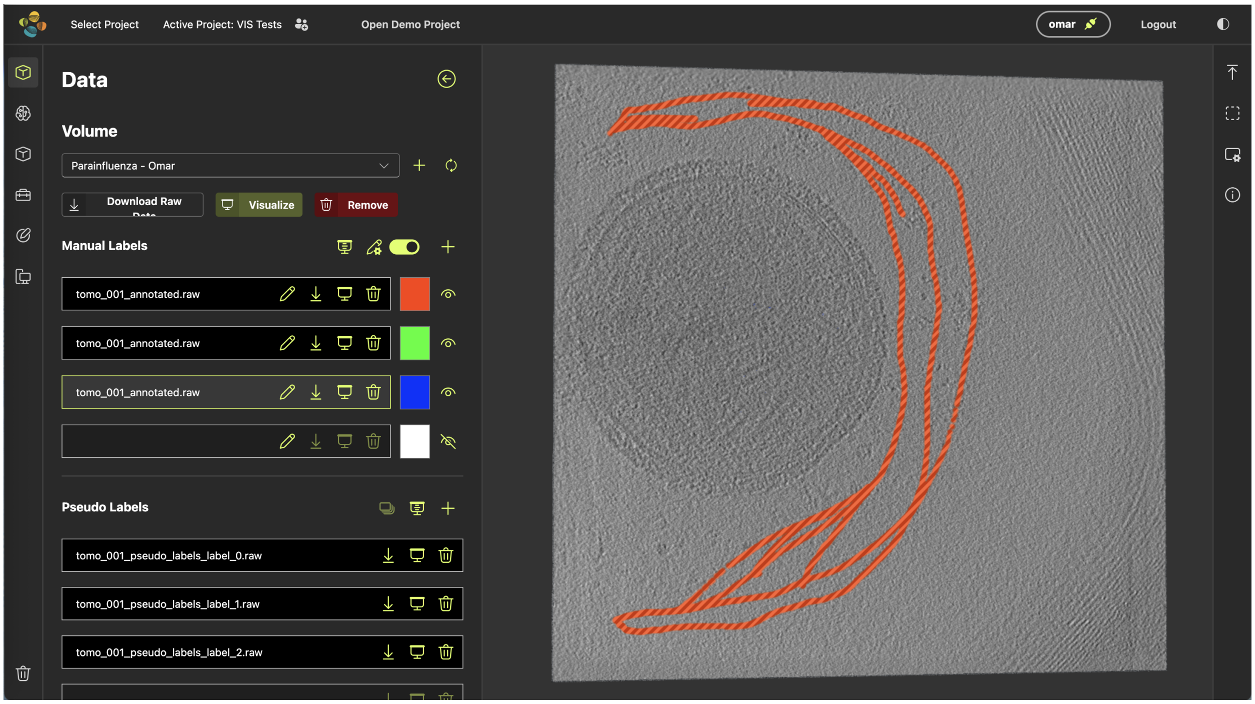

Figure 3: Manual annotation of tomogram's background

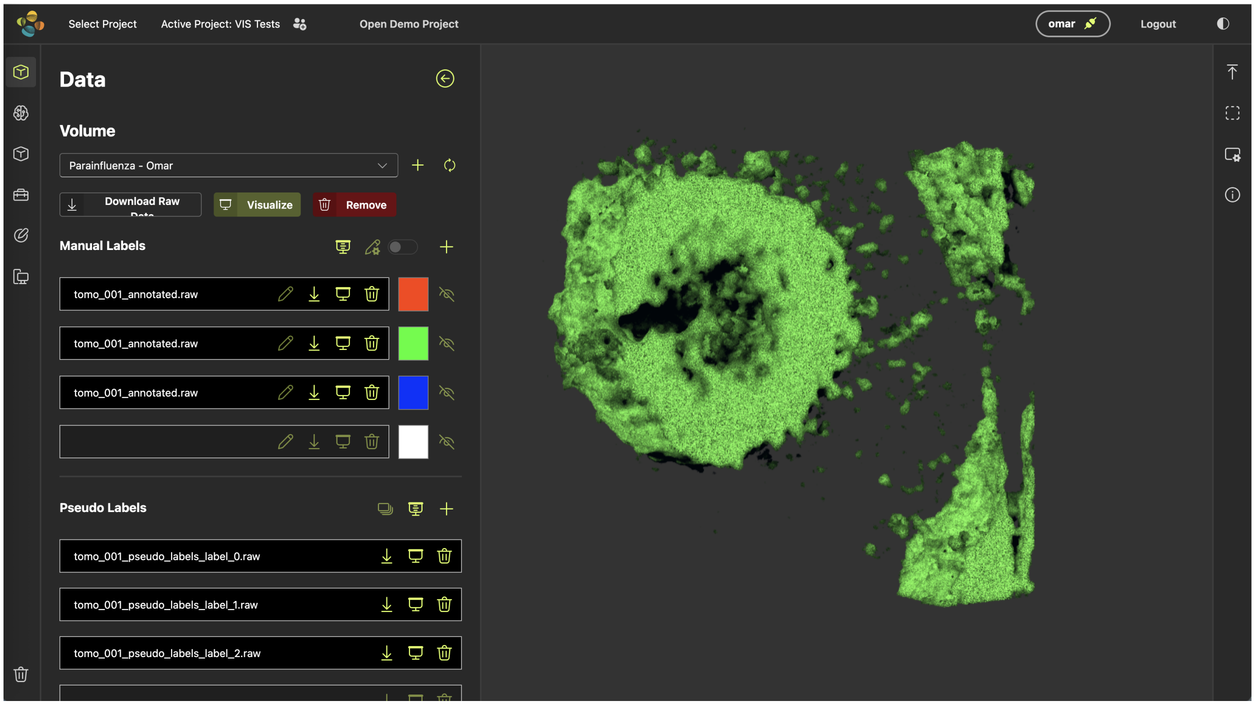

Figure 4: Pseudo-labeling of Parainfluenza Virus; only virus

Subsequently, they propagated these initial annotations using Ilastik, resulting in two important pseudo-labeled volumes as shown in: ?? a central "hole"-shaped label representing a significant background area, and ?? a complementary "sphere-like" label encapsulating the entirety of the virus particle.

Additionally, the team successfully demonstrated the system's collaborative strengths by concurrently accessing and visualizing the processed tomogram from three separate client computers connected to the same local network. Each machine downloaded and displayed extensive data without any performance degradation, enabling team members to assess segmentation quality together. This collaborative scenario demonstrates the framework's scalability and highlights its practical advantages for efficiently sharing, visualizing, and analyzing substantial volumetric datasets.

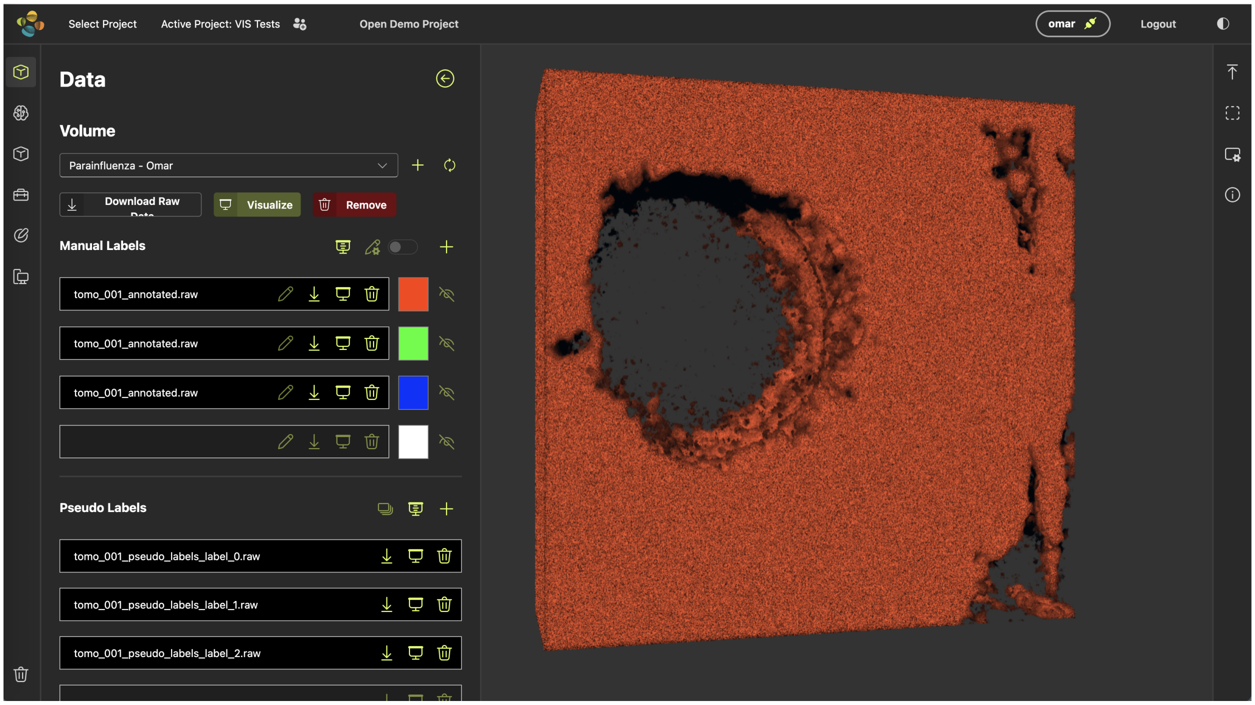

Figure 5: Pseudo-labeling of Parainfluenza Virus; only background
